# Suitability of the Openly Accessible 3D Printed Prosthetic Hands for War-Wounded Children

**DOI:** 10.3389/frobt.2020.594196

**Published:** 2021-01-11

**Authors:** John-John Cabibihan, Farah Alkhatib, Mohammed Mudassir, Laurent A. Lambert, Osama S. Al-Kwifi, Khaled Diab, Elsadig Mahdi

**Affiliations:** ^1^Department of Mechanical and Industrial Engineering, Qatar University, Doha, Qatar; ^2^School of Mechanical Engineering, University of Western Australia, Perth, WA, Australia; ^3^School of Public Administration and Development Economics, Doha Institute for Graduate Studies, Doha, Qatar; ^4^Department of Management and Marketing, Qatar University, Doha, Qatar; ^5^Qatar Red Crescent Society, Doha, Qatar

**Keywords:** prosthetics, assistive technologies, war-wounded, 3D printing, grasping

## Abstract

The field of rehabilitation and assistive devices is being disrupted by innovations in desktop 3D printers and open-source designs. For upper limb prosthetics, those technologies have demonstrated a strong potential to aid those with missing hands. However, there are basic interfacing issues that need to be addressed for long term usage. The functionality, durability, and the price need to be considered especially for those in difficult living conditions. We evaluated the most popular designs of body-powered, 3D printed prosthetic hands. We selected a representative sample and evaluated its suitability for its grasping postures, durability, and cost. The prosthetic hand can perform three grasping postures out of the 33 grasps that a human hand can do. This corresponds to grasping objects similar to a coin, a golf ball, and a credit card. Results showed that the material used in the hand and the cables can withstand a 22 N normal grasping force, which is acceptable based on standards for accessibility design. The cost model showed that a 3D printed hand could be produced for as low as $19. For the benefit of children with congenital missing limbs and for the war-wounded, the results can serve as a baseline study to advance the development of prosthetic hands that are functional yet low-cost.

## 1. Introduction

The loss of upper limbs has significant impact on the functional activities and social interactions of a person. The loss of upper limbs can be classified according to congenital limb loss or acquired limb loss. There is a 2:1 incidence ratio of congenital limb loss to acquired limb loss (Masada et al., 1986; Vannah et al., 1999; Vasluian et al., 2013). Congenital limb loss is attributed to malformations that have structural abnormalities of prenatal origin (Czeizel, 2005). The prevalence of upper limb loss is twice that of the lower limbs (Hirons et al., 1991). Acquired limb loss can be due to various reasons, including diseases or traumatic amputations from machinery, vehicular accidents, electrical injuries, or weaponry (Krebs et al., 1991).

In recent years, the acquired loss of the upper limbs have further increased due to warfare. Children are the most vulnerable victims of wars. Like other civilians, they can suffer a range of war-related injuries. Improvised explosive devices (IEDs), landmines, mortars, and air strikes are more likely to kill or permanently impair a child due to their inclination for outdoor activities. In 2017 alone, the United Nations General Assembly Security Council (2018) reported that there were around 9,624 children who were killed or maimed in armed conflicts worldwide (Table 1).

**Table 1:** Number of children affected worldwide by armed conflicts in 2017 (Adapted from United Nations General Assembly Security Council, 2018).

**Country**	**Killed**	**Maimed**	**Total**
Afghanistan	861	2,318	3,179
Central African Republic	61	43	104
Columbia	18	35	53
Democratic Republic of Congo	156	178	334
Iraq	279	438	717
Israel and State of Palestine	15	1,165	1,180
Lebanon	8	12	20
Libya	40	38	78
Mali	19	15	34
Myanmar	196	24	220
Somalia^*^	–	–	931
South Sudan	36	57	93
Sudan	19	75	94
Syrian Arab Republic	910	361	1,271
Yemen	552	764	1,316
Total	3,170	5,523	9,624

* *Data for killed or maimed were not provided*.

In the Syrian Civil War (2011-present), Handicap International (Bevington, 2015) estimated that one million people were injured and around 8% of them require prosthesis or orthosis. That translates to a latent demand of around 80,000 individuals who need such devices in one country alone. The vulnerability of the war-wounded is usually worsened by the collapse of the healthcare system. The Physicians for Human Rights (2015) have documented the systematic attacks on healthcare providers in Syria. To compensate for the lack of healthcare services, Qatar Red Crescent Society (a member of the Red Cross Red Crescent Societies), the International Committee of the Red Cross, Humanity and Inclusion (formerly Handicap International), and Médecins Sans Frontières (MSF) have come to the forefront of humanitarian assistance. For them, however, the provision of prosthetic limbs has become problematic because of the prohibitive prices in the context of international donor fatigue.

The price of commercially-available body-powered prostheses ranges from $4,000 to $10,000 (Resnik et al., 2012; ten Kate et al., 2017) while the electrically-powered ones cost between $25,000 and 75,000 (Resnik et al., 2012; van der Riet et al., 2013; ten Kate et al., 2017). For government-compliant upper extremity prosthesis, the American Orthotic and Prosthetic Association (2015) estimated that the price was between $1,500 and 5,000. All these amounts render the purchase of a prosthesis unaffordable for most of those who live in difficult living conditions, such as in the war zones, refugee camps, or low-income countries. The statistics in Table 1 are miniscule as compared to the demand for mass-produced consumer goods like mobile phones or athletic shoes. Due to the various levels of limb loss or amputations among the patients and the various preferences for functionality or other features (Korkmaz et al., 2012), a mass production approach for prosthetics is not feasible. There is patient-specificity for each prosthetic device.

An emerging technology for the fast production of low-cost prosthetics is three-dimensional (3D) printing (Cabibihan et al., 2015; Cabibihan et al., 2018; Alhaddad et al., 2017; Alturkistani et al., 2020). The 3D printing process is the additive deposition of material in a layer-by-layer manner to construct parts from a 3D computer-aided design (CAD) model (Hull, 1986). Consumer-grade desktop 3D printers, cost between $250 and 2,500. There are advantages of using 3D printing for prosthesis fabrication. First, the process does not need the numerous constraints imposed by changing the tools and switching manufacturing processes for each part. Secondly, 3D printing allows free-form shape, which can replicate the contours of human limbs. It allows the fabrication of prosthesis that is specific to the shape and size of each patient. Lastly, because the fabrication of parts is at low volume, the inventory of parts is minimized, thus, further minimizing the production costs.

In this paper, we ask whether the openly accessible, body-powered 3D printed prosthetic hands are suitable for the use of children (i.e., under 18 years old) with missing hands in low-resource settings. First, we evaluated all the published designs of openly accessible 3D printed prosthetic hands for their suitability to those with congenital loss of hands or war-related amputation. Next, we investigated the grasping postures of a representative design of a prosthetic hand. There were a few available designs but their cable-driven mechanisms and the materials used for 3D printing were similar. Third, we investigated the probable design aspects where failure can occur: the cables could break and the grasp could become compromised, the material in the fingers could break due to the high stresses from the cables that were under tensile forces, or the fingers' joints could fail due to the cyclic loads during the grasping and carrying of objects. Fourth, we developed a cost model to approximate the minimum price of each 3D printed hand. Lastly, we discussed the implications of this work for children with congenital limb loss and the war-wounded.

## 2. Openly Accessible 3D Printed Prosthetic Hands

As a baseline study, we investigated the body-powered 3D printed hands that were available online. Prosthetic hands that were controlled using pattern recognition of electromyographic (EMG) signals and other sensory feedback strategies were excluded in the investigation (Kuiken et al., 2016; Resnik et al., 2019).

Some of the available designs are shown in Table 2. These hands are anthropomorphic consisting of five fingers, each featuring two or three phalanges. One joint links the wrist to the harness, which is mounted to the stump of the amputated part of the arm. These types of mechanisms are considered as underactuated because the number of degrees of actuation is lower than the degrees of freedom (DOFs) on the whole mechanism (Birglen et al., 2008). For the hands in Table 2, there are at least 10 DOFs and a single mode of actuation, which is the flexing of the joint between the wrist and the harness.

**Table 2:** Openly accessible 3D printed prosthetic hands: structural material and the types of cables for flexion and extension.

**Prosthetic hand**	**Design**	**Joints**	**Material**	**Flexion**	**Extension**
Cyborg Beast (Zuniga, 2015)	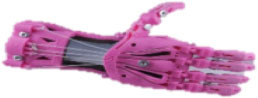	10	ABS^a^ or PLA^b^	Non-elastic cables	Elastic cords
Falcon Hand (Arabian, 2014)	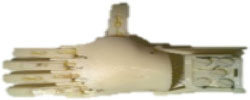	11	ABS	Non-elastic cables	Orthodontic rubber bands
FlexyHand (Wood, 2014)	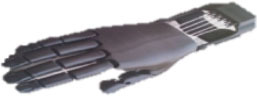	14	PLA or Filaflex	Non-elastic cables	Flexible joints
K1 Hand (Keuster, 2015)	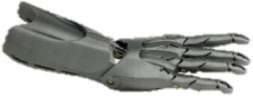	14	ABS or PLA	Non-elastic cables	Elastic cords
Phoenix Hand (Bryant, 2016)	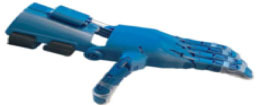	10	ABS or PLA	Non-elastic cables	Elastic cords
Raptor Reloaded (e-NABLE Community, 2014)	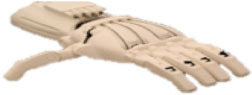	10	PLA	Non-elastic cables	Elastic cords

a *Acrylonitrile butadiene styrene*.

b *Polylactic acid*.

The fingers are actuated by the cables that are connected to the wrist. To control the body-powered prosthetic limb, cables are used to transfer the movements exerted from the body part to the prosthesis. This movement could be from the chest, shoulder, elbow, or wrist depending on the level of amputation. The flexion of the fingers depends on the tension force of the non-elastic cables, while the extension of the fingers depends on the restorative effect of the elastic cord that has a certain amount of flexibility, which then allows the return of the fingers to their natural pose (Alkhatib et al., 2019b).

**Figure 1:**
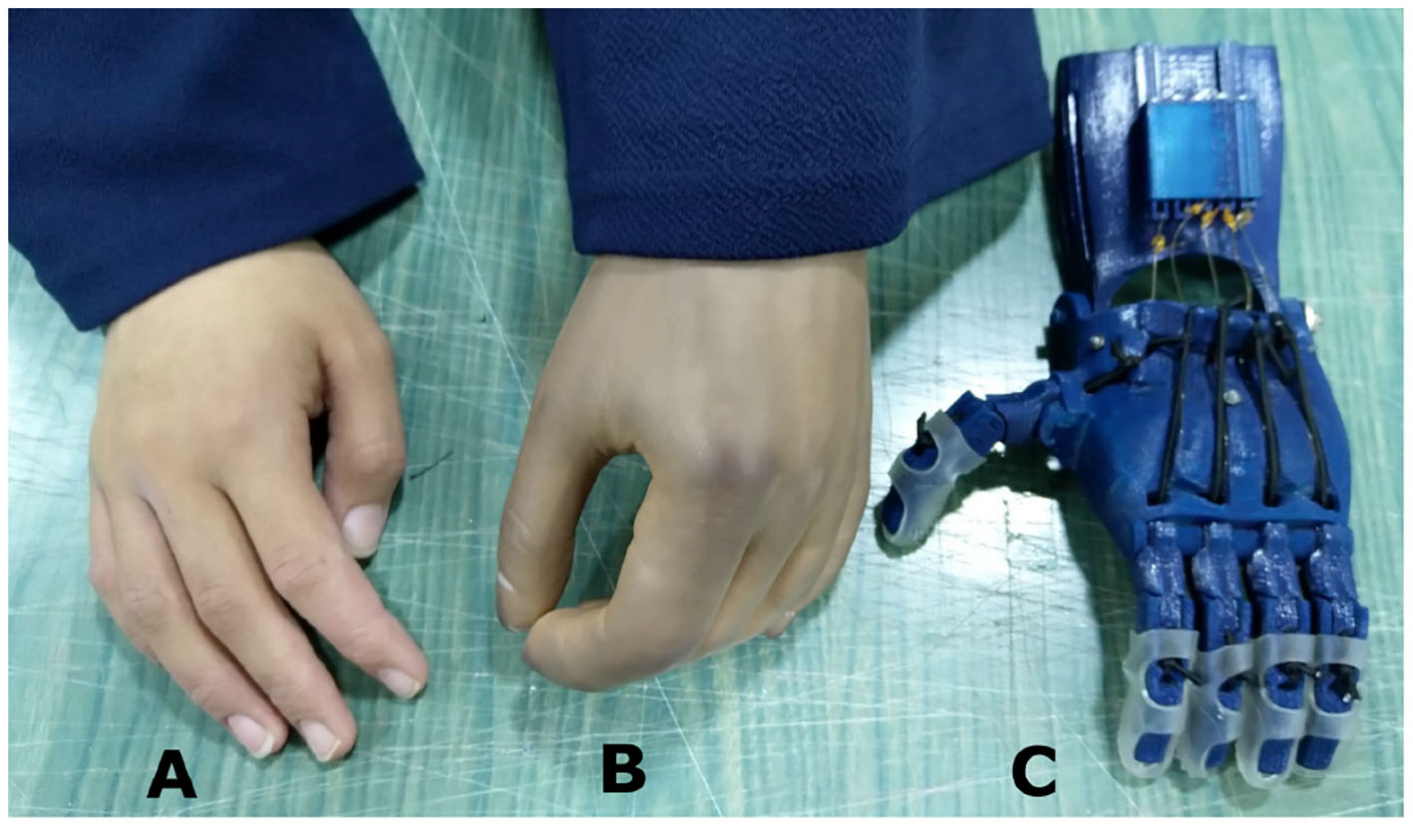
An amputee with a cosmetic prosthetic hand in one of our field interviews. **(A)** The non-affected hand. **(B)** A cosmetic hand with a darkened complexion due to the aging of the silicone material and smudging from dark clothes. **(C)** The Raptor Reloaded 3D printed prosthetic hand as a representative design of openly accessible 3D printed hands.

Among these designs, the Raptor Reloaded Hand (Figure 1) from the e-NABLE community has proven to be popular and is currently being used by more than 1,500 amputees from 40 countries because of its simple assembly and fairly acceptable appearance (Owen, 2017). This design has been reported in previous works (Arabian et al., 2016; Burn et al., 2016; Greene et al., 2016; Sullivan et al., 2017; ten Kate et al., 2017; Vujaklija and Farina, 2018). Further studies are needed to evaluate the technical integrity and functionality of this hand. We used this design to evaluate the movement, grasping forces, failure modes, and associated costs to produce a prosthetic hand for those with congenital limb loss or for the war-wounded.

## 3. Materials and Methods

### 3.1. Grasping Poses

The human hand is capable of various grasp types. It is capable for full-hand grasping (i.e., power grasps) or for dexterous grasping (i.e., precision grasps) of various objects. There are 15 joints and 20 DOFs in the human hand (Jones and Lederman, 2006; Kapandji, 2016). The human hand has been shown to perform 33 grasp postures (Feix et al., 2013; Feix et al., 2016). In that study, the 33 grasps were achieved using 17 objects. Among those objects were a ball, a coin, cylinders of various diameters, and others that are representative of objects in daily life. The full list of grasp postures can be found at the link.

#### 3.1.1. Materials

The Raptor Reloaded 3D design was downloaded and was used at the default scale of the original file (e-NABLE Community, 2014; Alkhatib et al., 2019a). The CAD file was 3D printed using Polylactic Acid (PLA; MakerBot, USA) filament on a desktop 3D Printer (Replicator 5th Generation, MakerBot Industries LLC, Brooklyn, NY, USA; build table: 29.5×19.5×16.5 cm^3^). The following settings were used: 215°C printing temperature, 0.2 mm layers, 2 shells, 35% infill, and the cooling fan was set to active mode. The printing was completed after 17 h. To complete the assembly, non-elastic and elastic cords were needed for the grasp and release mechanism. The non-elastic cables (super Dyneema strong braided fishing line, SeaKnight, China) were required to flex the fingers. Elastic cords (3 mm dia, Polypropylene Shock Cord, Sgt. Knots Supply Co, NC, USA) were used to return the fingers to their default pose. A knotting technique, known as the improved clinch knot, was used to firmly secure the cables and cords.

#### 3.1.2. Selection of Grasp Poses

The prosthetic hand is a transcarpal prosthetic hand. As such, a user dons the prosthetic hand and has to flex the wrist so that the grasping can be done. The protocol to find the grasping set was conducted as follows. First, a healthy child (8 years old) wore the transcarpal prosthetic hand through straps. The straps within the prosthetic hand simulated the grasping of a child amputee. Second, the images of the 33 grasps (Feix et al., 2016) were displayed on the screen, which the child repeated. In accordance to the procedure in Deimel and Brock (2016), the last step was to judge the quality of the grasp by moving the grasped object. Three consecutive trials were done for repeatability. We then shortlisted the grasp poses that the prosthetic hand was capable of.

### 3.2. Grasping Range of Motion

For an underactuated hand, all fingers wrap around the surface of an object. In cases where an object is smaller than the enclosing volume of the fingers, the fingers that are not touching the object will continue to flex until the structural limits are reached. For the representative sample (i.e., the Raptor Reloaded Hand), we investigated the limits imposed by the structural constraints. In this section, we asked whether the range of motion of the fingers was similar to that of the human hand. Additionally, we wanted to know how much flexion force on the wrist was required to achieve the prosthetic hand's range of motion.

#### 3.2.1. Data Analysis

The positions of the fingertip were determined according to its *X* and *Y* coordinates. A geometrical scheme was then developed to understand the grasping relationship between the finger's joints and links with its geometry. Forward kinematics was carried out by determining the Denavit-Hartenberg (D-H) parameters (Corke, 2017).

**Figure 2:**
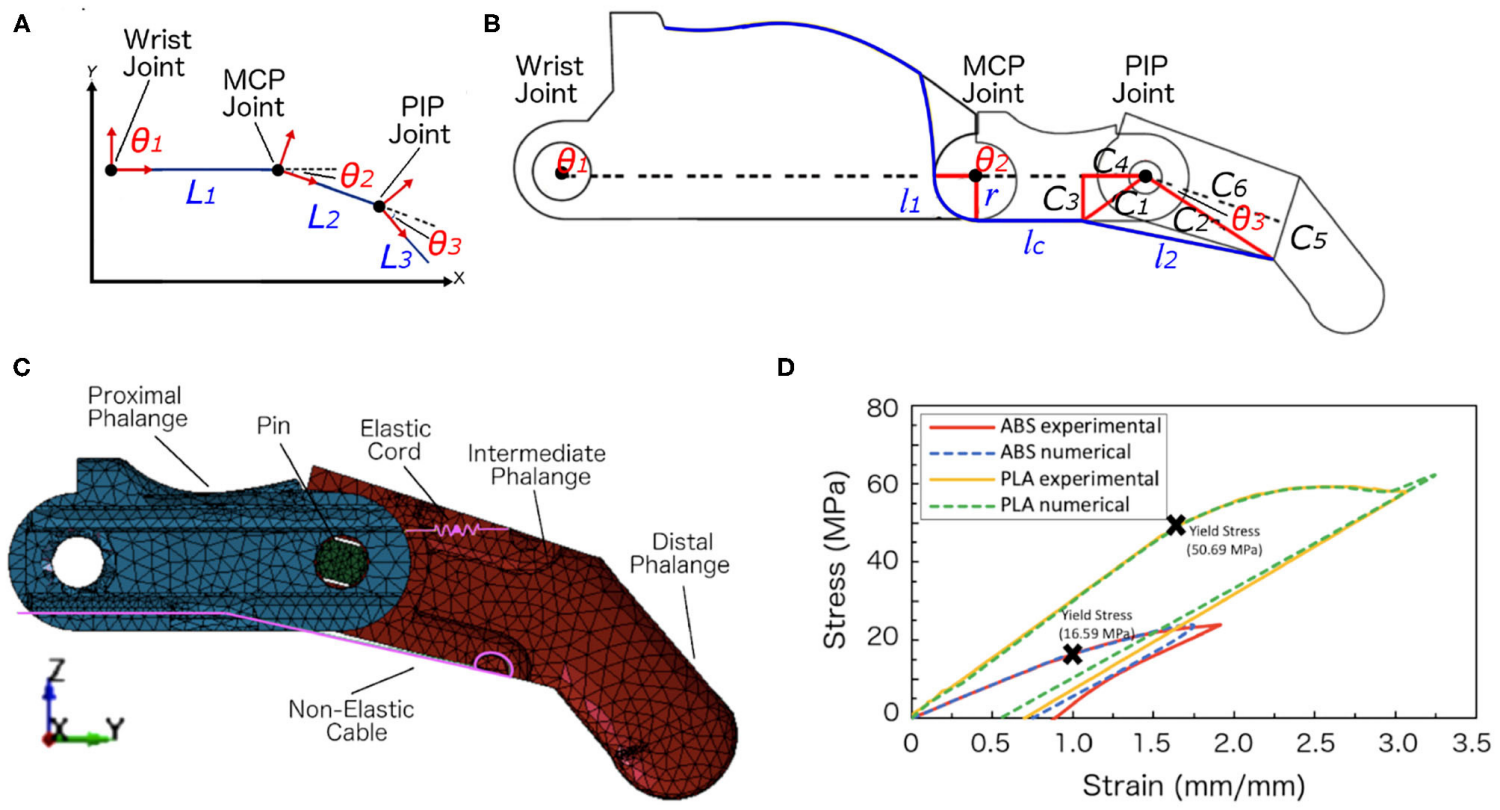
Schematic illustrations, meshed model, and materials characterization. **(A)** The three links of the index finger showing the three local coordinates and the variables. **(B)** The geometrical representation of the finger mechanism showing all the variables to calculate the final position of the fingertip. **(C)** The finite element model of the index finger. The model included two finger phalanges and the pin at the PIP joint. The non-elastic cables and elastic cords were embedded within the structure. **(D)** The experimental and numerical stress-strain curves of the ABS and PLA filament materials that were obtained from the tensile tests that we conducted. The yield stresses were marked for the two materials.

Figure 2A shows the link frame of the index finger. The two-dimensional Cartesian coordinates system (x,y) defines the origin point (0,0) at the wrist joint where θ_1_ = 0. The D-H convention was used to create the transformation matrices based on four parameters, which can be obtained from the link frame of the prosthetic hand. These parameters are the link lengths, *a*_*i*_, link twists, α_*i*_, link offsets, *d*_*i*_, and joint angles, θ_*i*_ (Table 3). The transformation matrices are shown in Equations (1)–(4).

(1) T01=(cosθ1−sinθ10L1cosθ1sinθ1cosθ10L1sinθ100100001)

(2) T12=(cosθ2−sinθ20L2cosθ2sinθ2cosθ20L2sinθ200100001)

(3) T23=(cosθ3−sinθ30L3cosθ3sinθ3cosθ30L3sinθ300100001)

(4) T03=T01T12T23

**Table 3:** Denavit-Hartenberg parameters of the 3D printed prosthetic index finger.

**Link number, *L*_*i*_**	**Link length, *a*_*i*_**	**Link twist, α_*i*_**	**Link offset, *d*_*i*_**	**Joint angle, θ_*i*_**
1	*L*_1_	0	0	θ_1_
2	*L*_2_	0	0	θ_2_
3	*L*_3_	0	0	θ_3_

The fingertip's posture can be expressed as position and orientation quantities, [*X, Y*, ϕ]^*T*^. The *X* and *Y* positions of the fingertip with respect to the wrist joint angle (θ_1_), metacarpophalangeal (MCP) joint angle (θ_2_) and the proximal interphalangeal (PIP) joint angle (θ_3_) were calculated by the following forward kinematics equations (Equations 5 and 6). The finger's orientation, ϕ, can be represented as the sum of the joint angles, θ_1_, θ_2_, and θ_3_ (Equation 7).

(5) $$\begin{array}{l} X=L_{1}\cos\theta_{1}+L_{2}\cos\left(\theta_{1}+\theta_{2}\right)+L_{3}\cos\left(\theta_{1}+\theta_{2}+\theta_{3}\right) \end{array}$$

(6) $$\begin{array}{l} Y=L_{1}\sin\theta_{1}+L_{2}\sin\left(\theta_{1}+\theta_{2}\right)+L_{3}\sin\left(\theta_{1}+\theta_{2}+\theta_{3}\right) \end{array}$$

(7) $$\begin{array}{l} \phi =\theta_{1}+\theta_{2}+\theta_{3} \end{array}$$

A mathematical relationship between the fingertip position and the applied grasping force was developed to calculate the *X* and *Y* positions of the fingertip. The geometry of the prosthetic index finger is shown in Figure 2B. The finger's grasping motion (flexion) was actuated by the tension of the cables, while the return motion (extension) was actuated by the elastic cords.

The flexion and extension resulted into changes in the MCP joint angle (θ_2_) and PIP joint angle (θ_3_). The Raptor Reloaded hand simplified the design by combining the DIP joint to the PIP joint. Henceforth, the DIP joint will not be mentioned. The value of these angles depended on the cable length, *l*_*Cable*_, which is in contact with the pulleys, the length of the cable along the phalanges, and the length until the fixed pin joint where the cable is attached (Equation 8). In other words, the more tension force is applied to the cable, the shorter the cable length will become. Consequently, more flexion will be achieved by the fingers. The cable's length has a maximum value at the natural pose ($\theta_{1}=\theta_{2}=\theta_{3}=0^{{}^{\circ}}$) and it has the minimum length at the full tension ($\theta_{1}=0^{{}^{\circ}};\theta_{2}=\theta_{3}=90^{{}^{\circ}}$). It is worth to mention that θ_3_ will never be zero in the actual design. The minimum value of θ_3_ can be assumed to be zero for simplification and this will not affect the finger analysis.

The cable length (*l*_*Cable*_) and the tension force applied on the cable (*F*_*tension*_) has a proportional relationship. This relationship was experimentally obtained by applying tension forces on the finger and measuring the values. A force gauge (DFS50, Nextech Global Company Limited, Thailand) was used for measuring the tension force and a Vernier caliper (part 530-118, Mitutoyo, Japan) was used for taking the length measurements.

The relationship between the cable's length and the MCP joint angle, θ_2_, and PIP joint angle, $\theta_{3}=0^{{}^{\circ}}$, can be calculated from Equations (9) to (12).

(8) $$\begin{array}{l} l_{Cable}=l_{1}+l_{2}+l_{c} \end{array}$$

(9) $$\begin{array}{l} l_{1}=r\left(\frac{\pi }{2}-\theta_{2}\right) \end{array}$$

(10) $$\begin{array}{l} l_{2}=\sqrt{C_{1}^{2}+C_{2}^{2}-2C_{1}C_{2}\cos\left(90^{{}^{\circ}}-\theta_{3}\right)} \end{array}$$

(11) $$\begin{array}{l} C_{1}=\sqrt{C_{3}^{2}+C_{4}^{2}} \end{array}$$

(12) $$\begin{array}{l} C_{2}=\sqrt{C_{5}^{2}+C_{6}^{2}} \end{array}$$

where *l*_*c*_ is a constant, while *l*_1_ and *l*_2_ are calculated from Equations (9) and (10), respectively. *C*_1_ and *C*_2_ can be obtained from actual measurements by using Equations (11) and (12). The variables *l*_*c*_, *C*_3_, *C*_4_, *C*_5_, *C*_6_, and *r* are constant lengths that can be measured directly from the design (Table 4). These constants will only be applicable if the downloaded design is not subjected to the scaling of the default dimensions.

**Table 4:** Design constants of the geometry as measured from the Raptor Reloaded Hand (dimensions in mm).

***l*_*c*_**	***C*_3_**	***C*_4_**	***C*_5_**	***C*_6_**	**r**
15.75	6.00	7.25	6.00	20.00	2.25

### 3.3. Finite Element Modeling

Non-linear finite element (FE) analysis was implemented using the software LS-DYNA (mmps R8.1.1, Livermore Software Technology Corporation, USA) to predict the maximum load applied on the prosthetic index finger before failure or breakage. The FE method divides the system into smaller parts (i.e., finite elements) and uses algorithms to solve the partial differential equations (PDEs). This numerical method approximates the system solution under the given initial and boundary conditions (Biddis et al., 2004; Mollica and Ambrosio, 2009). The FE method was earlier used in the analysis of prosthetic fingers and structures (Cabibihan et al., 2006a,b, 2014). In the current work, the locations with potentially high stress concentration were predicted to be at the distal finger phalange, proximal finger phalange, and at the pin. An FE model was created to determine the stresses at the critical components of the prosthetic hand. The various conditions and assumptions are described henceforth.

#### 3.3.1. Geometry

The open-source CAD files were downloaded from e-NABLE Community (2014), and the original design was modeled as it is. The model included the proximal phalange, the combined intermediate and distal phalanges, the non-elastic cable, the elastic cord, and the pin at the PIP joint (Figure 2C). For the purpose of saving computational time, the wrist, palm, and the pin at the MCP joint were not modeled since the direct contact with the objects comes from the distal and proximal finger phalanges.

#### 3.3.2. Geometry Meshing

To create the FE model, the finger geometry was subdivided into small 3D quadratic tetrahedron solid elements. Each element has four nodes and one nodal rotation to eliminate the probability of rotational deformation. For the non-elastic cable, the beam elements were used to model the cable because it has constant cross-sectional properties, its length is larger than its width, and it handles a load, which is distributed along its length. The beam elements consisted of three nodes in three-dimensional space. Two nodes were used for the identification of the geometry and the third node was for the orientation of the beam element. To model the elastic cords, one discrete element was used with one degree of freedom and two nodes. This discrete element has a spring behavior to simulate the elasticity of the elastic cord. A spring constant of 1,000 N/m was assumed, based on the elastic linear relationship between the force applied and displacement created (Hooke's law). In our study the applied force did not exceed 25 N and the extended displacements were relatively small (measured in mm), thus the spring constant was assumed on average.

H-refinement test was used to conduct the convergence study. With this process, the number of elements were increased in the model by reducing the element size. The initial mesh size ranged between 2.0 and 2.4 mm. The maximum value of von Mises stress was selected with respect to the number of elements. For computational time savings and because the maximum von Mises stress was almost constant after having 53,198 elements, we used the current model, with the element size ranging between 1.2 and 1.8 mm.

#### 3.3.3. Materials

Two types of filament materials were evaluated in the analysis: Acrylonitrile Butadiene Styrene (ABS; MakerBot ABS) and Polylactic Acid (PLA; MakerBot PLA). Both materials were compatible with the 3D printer (MakerBot Industries LLC, NY, USA). The piecewise linear plasticity material model (MAT_024) (Hallquist, 1993) from LS-DYNA material library was used to model the ABS and PLA distal finger phalange, the combined intermediate and proximal finger phalange, and the pin. The modeled non-elastic cable was a braided fishing line cable (super Dyneema strong braided fishing line, SeaKnight, China). The plastic kinematic material model (MAT_003) was used to model the non-elastic cable with very low strain rate because the cable was assumed to have no deformation with respect to time.

#### 3.3.4. Materials Verification

To verify the selected material model MAT_024, experimental tensile tests for ABS and PLA filaments were simulated numerically using LS-DYNA. The experimental tensile tests were performed using a universal testing machine (5969 Series Universal Testing Systems, Instron, USA). The loading rate for the tensile test was set to 5 mm/min. The samples of ABS and PLA materials were printed according to ASTM D638 standard (ASTM D638-14, 2014) and the same printer and printing conditions were used as described in section 3.1.1. Table 5 shows the obtained material properties of the two filament materials from our experimental test.

**Table 5:** Mechanical properties obtained experimentally from the tested 3D printed ABS and PLA samples.

**Material**	**Mass density (g/cm^**3**^)**	**Young's modulus (GPa)**	**Ult. tensile stress (MPa)**	**Failure strain (%)**
ABS	1.10	1.40	32.00	1.05
PLA	1.30	3.90	54.00	2.20

Elasto-plastic materials were defined as materials that achieve their elastic and plastic behaviors after reaching the yield stress of the material. On the contrary, metals undergo plastic deformation after reaching their yield stresses. The resulting stress-strain curves from the experimental tests were used to define the effective plastic strain of the material. The effective plastic strain is a value that increases whenever the material is actively yielding. This value was calculated incrementally over a period of time to characterize the plastic deformation. Figure 2D showed good agreement in the stress-strain curves between the experimental and numerical results. The yield stresses obtained from the experimental tensile tests for the ABS and PLA materials were 16.59 and 50.69 MPa, respectively.

#### 3.3.5. Boundary Conditions

Three important conditions were taken into account in modeling the prosthetic finger. First, the hole at the proximal phalange was supported in all directions (i.e., in translation and rotation), with the exception of the rotational movement around the x-axis. Second, the pin was fully supported in all directions (i.e., all the degrees of freedom of the pin nodes were constrained). Third, no support was applied on the intermediate phalange, which means that it was free to move in any direction. To constrain the intermediate phalange, a frictional contact between the pin nodes and intermediate phalange node was applied with a friction constant of 0.3. The same contact was applied between the proximal phalange and the intermediate phalange.

#### 3.3.6. Loading

Tension force was applied on one node of the cable. The ABS finger model was subjected to 5 and 15 N, while the PLA finger model was subjected to 5, 15, and 25 N. These values were close to the 22.2 N maximum force limit to single-handedly grasp, pinch, or twist objects (Standards for Accessible Design; United States Department of Justice, 2010; std. no. 309.4).

### 3.4. Production Cost Analysis

In evaluating the cost for each 3D printed hand, the following components were considered: the equipment cost (i.e., 3D printer), material cost (i.e., filament, cables, and elastic cords), labor cost of the technician, the cost of maintaining the 3D printer, and the energy cost (Table 6). The equipment, filament, and maintenance costs were obtained from the manufacturer of the 3D printer (Replicator+, MakerBot Industries LLC, NY, USA). The cable (super Dyneema strong braided fishing line, SeaKnight, China) and elastic cord Polypropylene Shock Cord, Sgt. Knots Supply Co, NC, USA) were sourced from industrial suppliers. The labor and energy costs were based on local costs in Doha, Qatar.

**Table 6:** Elements and prices for the cost model calculations.

**Cost model element**	**Item**	**Unit cost**	**Note**
Equipment cost	3D Printer	$2,800/unit	10,000 h life expectancy (approx.)
Material cost	PLA Filament	$43/kg	130 g/hand
	Cables	$15/spool of 500 m	0.625 m/hand
	Elastic cords	$9/spool of 50 m	0.5 m/hand
Labor cost	Technician's Salary	$1,000/month	Monthly part-time salary
Maintenance cost	3D Printer Extruder	$200/unit	5,000 h life expectancy (approx.)
Energy cost	Power	$0.47/kWh	167 W; 17 h printing/hand

The life expectancy of the 3D printer was estimated to be 10,000 h or around 3.5 years. The extruder was approximated to be replaced at the half life expectancy of the 3D printer. The labor cost came from the university's salary guidelines and the energy cost was based on the electricity consumption of the machine where the unit price was based on the data provided by the local energy supplier.

The total cost to produce one 3D printed hand consisted of the following cost components:

(13) $$\begin{array}{l} C=C_{EQ}+C_{RM}+C_{LA}+C_{MA}+C_{EN} \end{array}$$

where *C*_*EQ*_ is the equipment cost per hour, *C*_*RM*_ is the raw material cost per hand, *C*_*LA*_ is the labor cost per hour, *C*_*MA*_ is the maintenance cost per hour, and *C*_*EN*_ is the energy cost per hour.

The equipment cost per hour was calculated as: *C*_*EQ*_ = (2, 800/10, 000) = *$*0.28/*hour*, which was based on the life expectancy of the 3D printer of 10,000 h and the initial equipment cost. The raw material cost for every printed hand was calculated as the sum of the filament ($5.59), non-elastic cable ($0.019), and elastic cord ($0.09) for a total of $5.70. The labor cost was calculated from the time to assemble the various parts of the printed hand. The assembly was around 1 h for each hand. The labor cost, *C*_*LA*_, is equal to:

(14) $$\begin{array}{l} C_{LA}=\frac{1,000\left(\frac{\$}{month}\right)}{8\left(\frac{hours}{day}\right)\times 5\left(\frac{days}{week}\right)\times 4\left(\frac{weeks}{month}\right)}=\frac{\$6.25}{hour} \end{array}$$

The maintenance cost per hour, *C*_*MA*_, was calculated based on a 2-year maintenance cost over the life expectancy of the 3D printer: *C*_*MA*_ = 400/10, 000 = *$*0.04/*hour*. The energy cost, *C*_*EN*_, is equal to $0.078 per hour. The total time to complete 3D printing of one hand was 17 h. The cost equation for producing one hand is as follows:

(15) $$\begin{array}{l} C=\left(0.28\times 17\right)+5.70+6.25+\left(0.04\times 17\right)+\left(0.078\times 17\right) \end{array}$$

## 4. Results

### 4.1. Limited Grasping Poses

**Figure 3:**
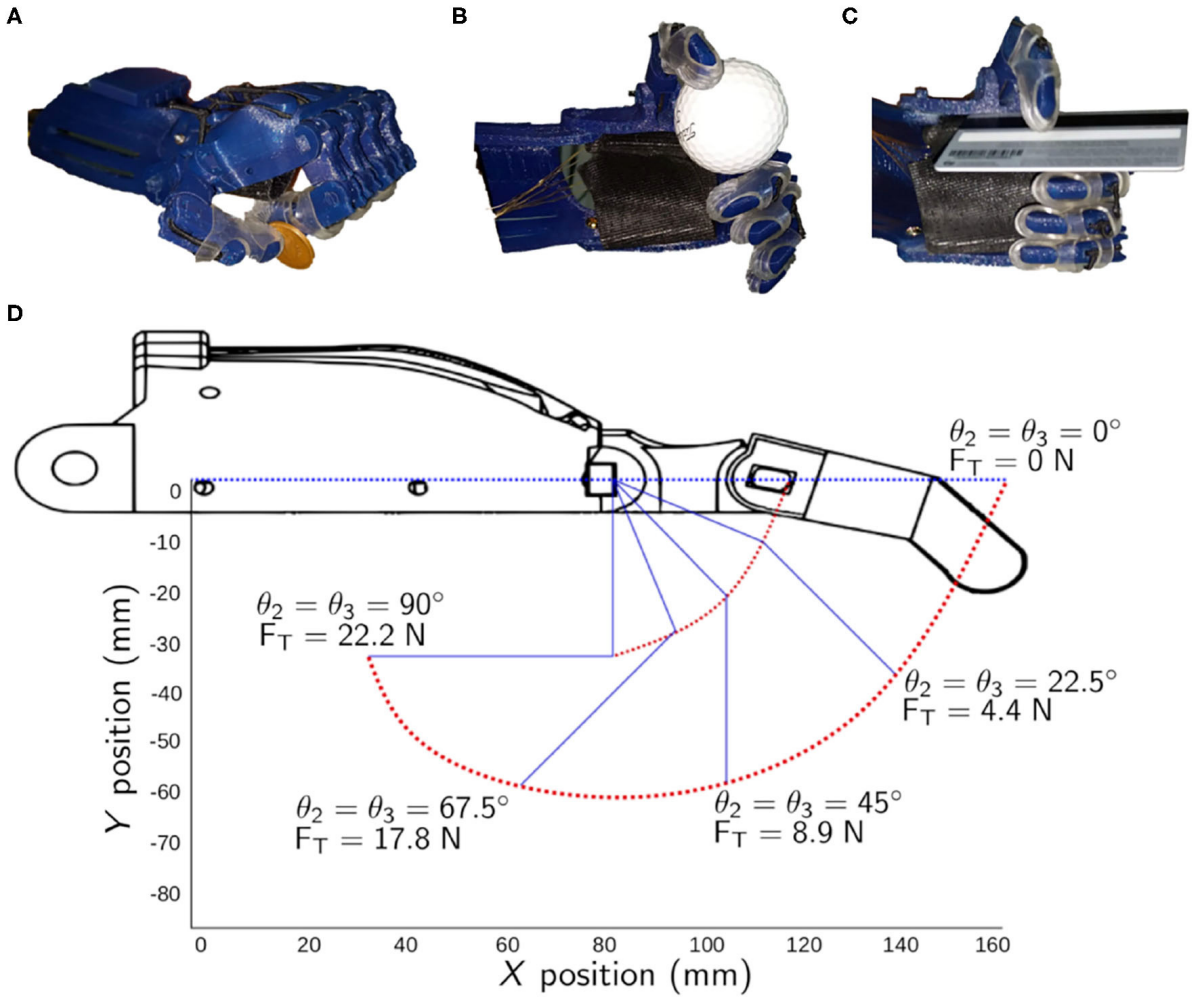
Grasping movements that can be achieved by the representative body-powered 3D printed prosthetic hand. **(A)** Palmar pinch. **(B)** Inferior pincer grasp. **(C)** Lateral grasp. **(D)** The positions of the index finger of the 3D printed prosthetic hand in the *X* and *Y* axes with respect to the MCP and PIP joint angles.

From the 33 total grasps that a human hand can do (cf. section 3.1), there were only three grasping postures that can be achieved using the 3D printed hand that was considered in this study (i.e., the Raptor Reloaded Hand). Figures 3A–C shows the grasp poses and representative objects: palmar pinch of a coin (7.7 g), lateral grasp of a credit card (10 g), and an inferior pincer grasp of a golf ball (46.4 g).

### 4.2. Cable Tension Analysis

The range of motion of the finger with respect to θ_1_, θ_2_, and θ_3_ are shown in Figure 3D. From the initial conditions, the joints θ_2_ and θ_3_ flexed to 22.5° when the cable applied a force of 4.4 N. The variable cable length, *l*_*VC*_, was only 1.2 mm (*l*_*VC*_ = *l*_*Cable,experimental*_ - 41.45, where 41.45 mm was the total cable length measured experimentally when $\theta_{1}=\theta_{2}=\theta_{3}=0^{{}^{\circ}}$). When a force of 8.9 N was applied, the joints θ_2_ and θ_3_ flexed to 45° and the variable cable length recorded was 5.7 mm. It took more tensile force to achieve a higher flexion angle. To flex both joints to 67.5°, a force of 17.8 N was required. The variable cable length to achieve that was 14 mm. The full flexion angle of 90° required that a user needs to exert a force of 22.2 N and an engagement of the cable to 15 mm in length. Table 7 compares the experimental total cable length from the theoretical total cable length calculated in Equation (8) and the experimental tests described earlier. The errors were calculated to be from 1.75 to 7.61%.

**Table 7:** Relationships between the grasping posture angles, forces, total cable lengths, errors, and the variable cable length.

**Angle, θ_2_ = θ_3_ (deg)**	**Cable tension force (N)**	**Total cable length, theoretical, (mm)**	**Total cable length, experimental, (mm)**	**Error **(%)****	**Variable cable length (mm)**
0	0	42.19	41.45	1.75	0
22.5	4.2	37.74	40.45	7.17	1.2
45	8.9	33.22	35.75	7.61	5.7
67.5	17.8	29.34	27.45	6.44	14.0
90	22.2	27.22	26.45	2.83	15.0

### 4.3. Failure Analysis

**Figure 4:**
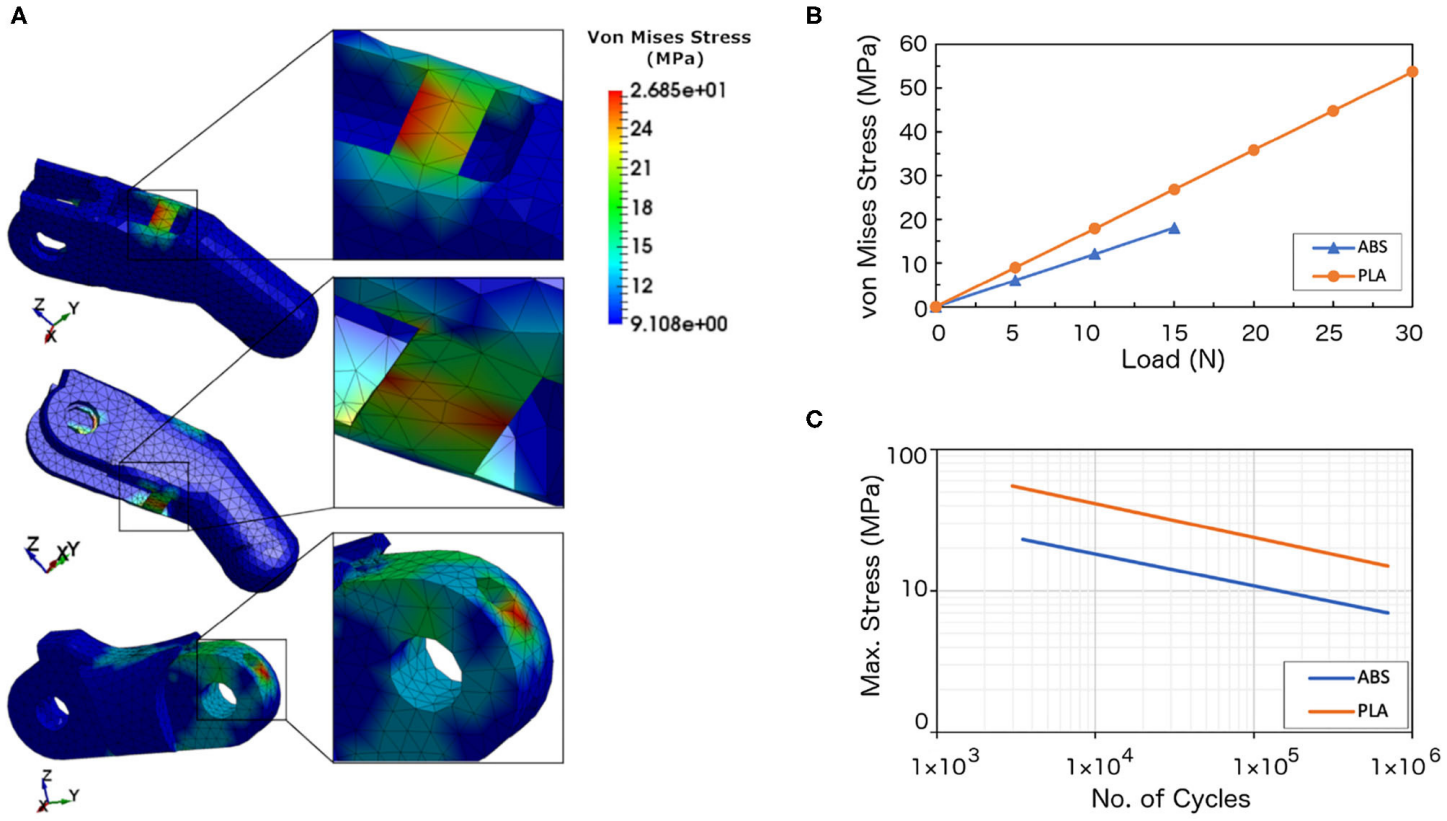
Analysis results. **(A)** Finite Element Analysis results showing the locations with the highest von Mises stress under applied loading from the cables being pulled. The maximum von Mises stresses were found at the top and bottom hinges and near the pin hole. These results are for the PLA finger model subjected 15 N loading. **(B)** Numerical results of the obtained von Mises stresses vs. applied load. **(C)** The endurance limit for ABS and PLA materials (Adapted from Caliskan et al., 2016; Ezeh and Susmel, 2019).

The yield stresses of the ABS and PLA materials were defined (Figure 2D). The yield point indicates the end of the elastic behavior and the start of plastic behavior of the materials (i.e., the finger will deform and fail beyond this value). Two different loads were applied on the ABS finger, and three loads on the PLA finger for investigating the material failure. Figure 4A shows the contour plots for the von Mises stresses of the PLA finger under 15 N loading (also see animations). It can be seen from the figure and animations that the highest stress concentration areas can be found at the hinges and at the pin holes in both the proximal and distal finger phalanges. From these results, it can be concluded that the initial failure can occur at these high stresses regions.

From the numerical results, the maximum stresses achieved at 10 N load for the ABS and at 25 N load for the PLA were 12.04 and 44.76 MPa, respectively. Figure 4B shows the von Mises stresses of the ABS and PLA with respect to the applied forces. The ABS material registered a maximum von Mises stress of 6.02 and 12.04 MPa when loaded with a 5 and 10 N forces, respectively. In comparison, the PLA material has higher maximum stresses as compared with the ABS material. The PLA have maximum stresses of 8.95, 26.85, and 44.76 MPa when loaded with forces of 5, 15, and 25 N, respectively. The von Mises criterion was used to determine whether the material will yield or fracture. If the value of the von Mises stress is equal or greater than the material's yield stress then the material will yield. As seen from Figure 4B, the stresses have increased linearly with the increase in the applied force. The estimated failure stresses were 13.75 N for the ABS material and 28.3 N for the PLA material.

When a 10 N load was applied to the PLA finger, the maximum stress obtained from the numerical analysis was 17.90 MPa (cf. Figure 4B). If we assume that the finger will have 50 cyclic movements per day, the finger will experience 18,250 cycles per year. Using the endurance limit for the PLA material (Figure 4C), this finger can withstand 3 × 10^5^ cycles before failure, which is equivalent to 16 years (Table 8).

**Table 8:** The life cycle of the ABS and PLA prosthetic fingers with respect to the load.

**Material**	**5 N**	**10 N**	**15 N**	**20 N**	**25 N**
ABS	1 × 10^6^ (no failure)	7 × 10^4^ (4 yrs)	Fail	Fail	Fail
PLA	5 × 10^6^ (no failure)	3 × 10^5^ (16 yrs)	7 × 10^4^ (4 yrs)	2 × 10^4^ (1 yr)	7.5 × 10^3^ (0.5 yr)

In the FE modeling, the tension force was applied to the non-elastic cable. Numerical results showed that the maximum stress obtained on the non-elastic cable was 100 MPa at 28 N of tension loading. The other stresses obtained at each load were tabulated in Table 9. However, from the product's specification sheet, the cable can hold up to 1,570 MPa at 440 N of tension loading. It can be concluded that no failure will occur at the cable unless there are other external conditions, such as tearing from the friction developed between the cable and the plastic material.

**Table 9:** Axial stresses from the applied load on the cable.

Applied load (N)	5	10	15	20	25	28
Axial stress (MPa)	17.8	35.7	53.6	71.4	89.3	100.0

### 4.4. Production Cost

The total cost for producing one unit of a 3D printed hand was calculated to be $18.72. The three major contributors to the total cost were the equipment cost per hour (*C*_*EQ*_), raw material cost per hand (*C*_*RM*_), and labor cost per hour (*C*_*LA*_). The maintenance cost per hour (*C*_*MA*_) and energy cost per hour (*C*_*EN*_) have minimal contribution to the overall cost. The *C*_*EQ*_ can be reduced by producing multiple hands at the same time (cf. Rickenbacher et al., 2013; Piili et al., 2015). The *C*_*RM*_ cost can be decreased by ordering large quantities directly from key suppliers.

## 5. Discussion and Conclusion

### 5.1. The Importance of 3D Printing for Prosthesis

The absence of limbs from congenital reasons or from warfare can have devastating physical, psychological and socio-economic consequences (Mckechnie and John, 2014; Griffet, 2016). For the war-wounded children, the consequences go beyond their impaired capacity to play, perform chores, and to care for oneself. Their loss of limbs can leave them with various social issues as well as mental disorders: post-traumatic stress disorders, generalized anxiety disorder, depression, and cognitive disorders (Betancourt et al., 2011; Hemmati et al., 2015). The thousands of children maimed by war each year have limited access to prosthesis services and it may take up to 10 years before a prosthetic limb can be fitted (Santa Barbara, 2006).

The emergence of 3D printing has opened many opportunities for artificial hands for assistive purposes (Tian et al., 2017; Negrello et al., 2020). This paper endeavored to answer whether the openly accessible designs of body-powered 3D printed prosthetic hands are suitable and affordable for the harsh environmental conditions of the war-wounded. There were four aspects that were evaluated: grasping poses, the range of motion of the grasps and the analysis of the corresponding cable lengths, the failure analysis in the various critical components of the 3D printed hand, and the cost of production.

### 5.2. Human vs. Prosthetic Hand: Differences in Grasping Movements

**Figure 5:**
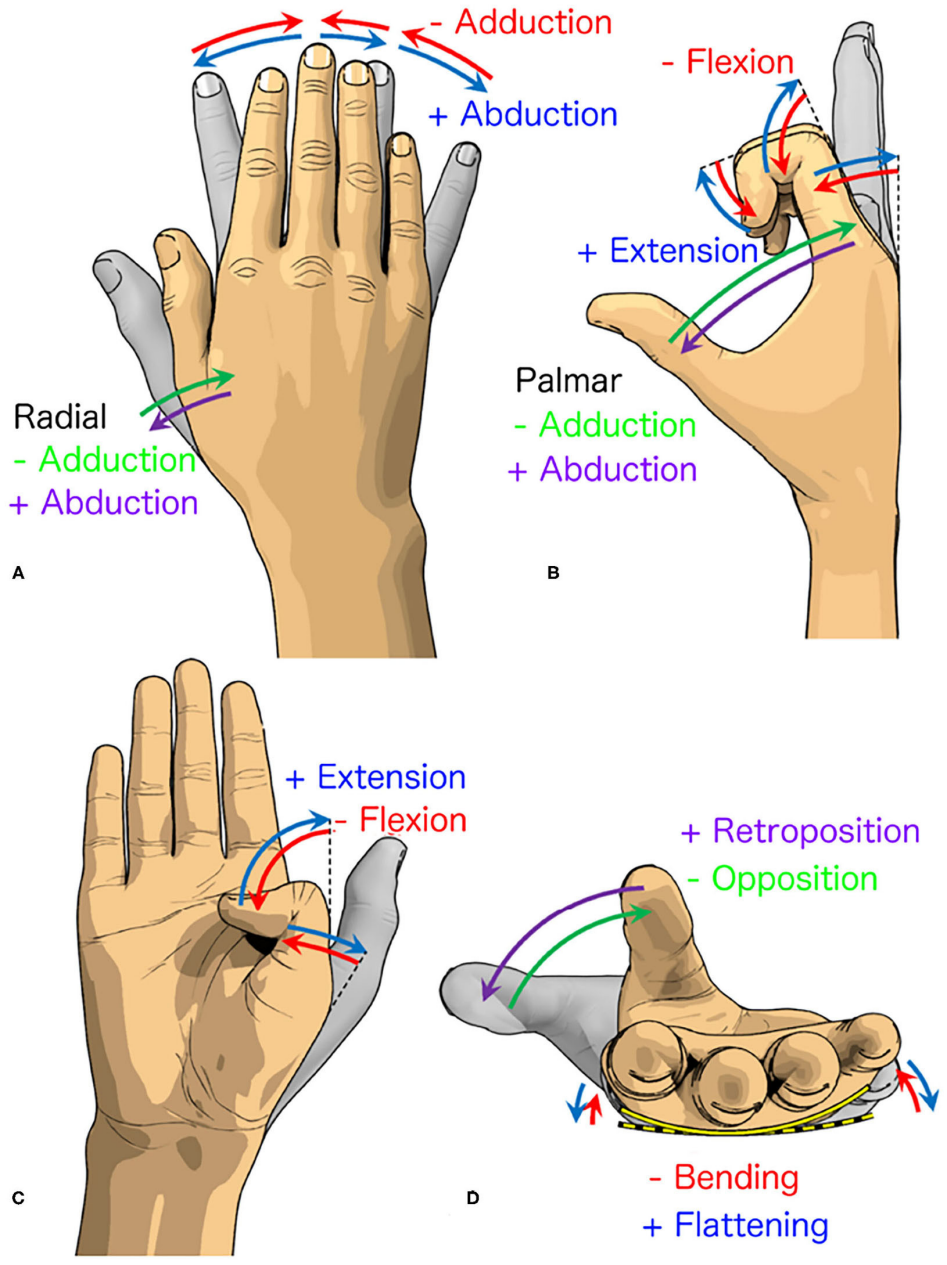
Various movements of the fingers. **(A)** The radial abduction/adduction of the thumb and the relative abduction/adduction of the remaining fingers. **(B)** The palmar abduction/adduction of the thumb and the flexion/extension of the remaining fingers. **(C)** The flexion/extension of the remaining thumb joints. **(D)** The opposition/retroposition of the thumb and the bending/flattening of the palm. The blue/purple and red/green arrows indicate the positive and negative directions, respectively.

The human hand can perform 33 grasp types due to the various combination of movements that it can do (Figure 5). The human hand is capable of the adduction/abduction of the five fingers with the radial adduction/abduction of the thumb, the flexion/extension of the five fingers with adduction/abduction of the palm, and the retroposition/opposition of the thumb with the bending/flattening of the palm. On the contrary, the fingers of the 3D printed hand are only capable of flexion and extension on a flat palm design. It is noteworthy to mention that the four fingers of the 3D printed hand can achieve the flexion and extension angles of up to 90° in both of the PIP and MCP joints, which are similar to the human hand (cf. Figure 3D). While the thumb can perform extension and flexion, the thumb is unable to perform retroposition/opposition in addition to the palm's inability to flex. The current 3D printed hand and similar prosthetic hand designs were limited to perform only 3 out of 33 grasps (i.e., palmar pinch, inferior pincer grasp and lateral grasp). The grasping postures of the investigated 3D printed hand were severely limited by the hand's structural design.

However, it may not be necessary to aim for a complete replication of the 33 grasps due to the cost constraints. An increase in the degrees of freedom and functionality has an implication on the increased complexity of the prosthetic hand. A complicated prosthetic hand can lead to an increase in the non-usage rate. In a future work, we can ask children with missing upper limbs on the priority of tasks that they wish they can do, a matter so far poorly taken care of in the literature. Thus, a balance for optimal design and affordable cost needs to be further investigated.

### 5.3. Robustness of 3D Printed Hands for Environments With Limited Resources

A mechanical apparatus that serves as a user's interface to various objects in the environment on a daily basis will tend to fail. A failure analysis of this interface (i.e., a prosthetic hand) becomes more relevant when the filament materials used for the 3D printing process are polymers. Ideally, prosthetic hands should be able to perform the basic grasping activities of daily living without failure.

Based on our results, the average life expectancy was found to be 4 years under light daily activities. Small, lightweight items like paper, ball, and cards are within the expected loading cycles (Table 8). Usually, such prostheses are designed for children who are still in the development stage and a continuous size upgrade is needed. Thus, the durability of these hands may not be crucial. Our results showed that the PLA material cannot be subjected to heavy loads (i.e., more than 28 N).

From a consumer psychology perspective, it can be argued that the repeated replacement of a device might decrease the trust in a device's functionality and reliability. To mitigate this issue, we anticipate to use a better structural materials, which can last longer and require less replacement. New materials with high strength (e.g., thermoplastic elastomers, nylon, polyvinyl alcohol) are expected to boost the confidence in this device. With improved designs of 3D printing extruders to process new materials, more improvements can be added to enhance the load capacity, grasping ability, and the appearance of this type of prosthetic hand.

The non-elastic cable's load capacity is high and is unlikely to fail (Table 9). On the other hand, the elastic cables are more likely to lose their elasticity with time, which makes the return motion slower or unreachable. The periodic replacement of these low-cost cables can solve this issue. The condition on the failure of the cables due to friction was not conducted. Simulating this condition will depend on the surface finish of the printed hand (i.e., related to the 3D printer's quality) and this cannot be considered it in our analysis because we would not be able to predict which 3D printer will be used by those in the war zones. Spare cables and elastic cords can be provided to the users so they can make the replacements when necessary.

### 5.4. Cost Considerations for Low-Resource Countries and Host Countries for Refugees

The design characteristics of conventional upper limb prosthesis are incompatible to the design requirements in locations where there is a lack of power supply, scarce resources, and zero options for warranties. The on-site production of prosthesis parts would significantly reduce the cost and time of shipping and delivery, and provide a higher level of accuracy.

The emergence of 3D scanning and printing is minimizing the dependence on highly-trained prosthetists in conflict zones. In the traditional prosthesis fabrication process, which rely on molding and casting, there needs to be some adjustments on fitting the resulting prosthesis to the amputee. The reason for that additional process was that the procedure to obtain the measurements was already flawed at the start. In the conventional process, the amputee would be asked to submerge the stump in gypsum plaster (plaster of Paris) or alginate. The stump, due to its compliant tissue, has already been deformed in the process (Cabibihan, 2011; Cabibihan et al., 2011). The ideal procedure is a non-contact way to obtain the data (i.e., 3D scanning). The 3D scanning approach is compatible with the 3D printing procedure.

In developed countries, the cost for conventional upper limb prostheses is from $1,500 to as high as $75,000. For such amount, there is the risk that the materials used in the prosthesis can be repurposed or bartered in case they are provided freely in conflict zones. With a basic 3D printed prosthetic hand costing as low as $19, prosthesis providers in developing countries and in those countries hosting refugees could find such options to be attractive.

### 5.5. Limitations and Future Work

The primary use of an upper limb prosthetic device is to let the user live without stigma. Both the prosthesis user and the people around the user give importance to the appearance of the prosthesis (Scotland and Galway, 1983). The current paper did not address the appearance of the prosthesis. Amidst a healthcare sector that is facing economic difficulties due to donor fatigue after almost a decade of conflict in areas like in Syria, the focus of the paper was in the technical evaluation of the benefits and limitations of the current 3D printed prosthetic hand designs. The current designs were intended to be affordable alternatives to the more expensive, traditional methods of manufacturing. Future work can address the fitting of a glove and its coloration.

In war-affected and low-resources countries, the main advantages of 3D printed prosthetic hands are in the portability of the 3D printers, the cost-effectiveness of the material, the possibility of on-site production, the amputee-specific design, and the low maintenance cost. These prosthetic hands are still not satisfactory for functional tasks for a user's daily activities and are not replacements for other improved and advanced designs. This type of prosthetic design and production technique must not be media-hyped because the users might expect too much. This is a temporary solution, but 3D printed prosthetics can still help the users in their basic daily activities and improve their self-confidence. With the exception of the motors, some of the elements we investigated here are also present in other mechatronic prosthetic hands. If resources are available and if the users so desire, further enhancements can be done by the inclusion of robotic elements in the prosthetic hand. The results presented herein serve as a baseline study to advance the development of prosthetic hands that are functional yet low-cost.

## Data Availability Statement

The original contributions presented in the study are included in the article/Supplementary Material, further inquiries can be directed to the corresponding author/s.

## Author Contributions

J-JC, FA, and EM conceived and designed the study. FA and MM performed the experiments. LL and KD arranged and conducted the field interviews and analyzed the users' feedback. OA-K developed the cost model. J-JC, FA, MM, and EM analyzed the data and interpreted the results. All authors wrote and contributed to the final version of the manuscript and approved the submission.

## Conflict of Interest

The authors declare that the research was conducted in the absence of any commercial or financial relationships that could be construed as a potential conflict of interest.
